# Adult-onset adrenoleukodystrophy presenting as a psychiatric disorder: MRI findings

**DOI:** 10.1590/S1980-57642012DN06040015

**Published:** 2012

**Authors:** Antonio Cézar Ribeiro Galvão, Gislaine Cristina Lopes Machado-Porto, Fábio Henrique de Gobbi Porto, Leandro Tavares Lucato, Ricardo Nitrini

**Affiliations:** 1 MD, PhD, Assistant Professor. Department of Neurology, Hospital das Clínicas of the University of São Paulo (HC/FMUSP), São Paulo SP, Brazil.; 2 MD, Department of Radiology, Hospital A.C. Camargo, São Paulo SP, Brazil.; 3 MD, Behavioral and Cognitive Neurology Unit, Department of Neurology, and Cognitive Disorders Reference Center (CEREDIC), HC/FMUSP.; 4 MD, PhD, Full Professor, Behavioral and Cognitive Neurology Unit, Department of Neurology, and CEREDIC, HC/FMUSP.

**Keywords:** leukoencephalopathies, leukodystrophies, adult-onset adrenoleukodystrophy, magnetic resonance imaging

## Abstract

A 35-year-old, previously healthy man presented psychiatric symptoms lasting four years, receiving treatment with neuroleptics. One year later he evolved with gait disequilibrium. After a further six months, cognitive symptoms were characterized with rapid evolution to a profound demented state. MRI showed signal changes in cerebral white matter and very long-chain fatty acids were detected in blood.

## Abstract (pt)

Homem de 35 anos, previamente saudável, iniciou há quatro anos sintomas psiquiátricos, recebendo tratamento com neurolépticos. Um ano após evoluiu com alterações do equilíbrio. Há seis meses apresentou distúrbios cognitivos, piorando rapidamente a um estado de profunda demência. RM do encéfalo revelou intensa alteração de sinal na substância branca cerebral e foram detectados ácidos graxos de cadeia muito longa no sangue.

## INTRODUCTION

Leukoencephalopathies encompass a heterogeneous group of disorders that involve CNS white matter. The cause of these conditions may be acquired or inherited. Acquired etiologies include inflammatory, vascular and neoplastic disorders, as well as infectious, vitamin deficiency and autoimmune diseases (including multiple sclerosis). Inherited leukoencephalopathies are called leukodystrophies. They are usually due to mutations in genes that encode protein components of the myelin membrane or enzymes implicated in the turnover of myelin.^1,2^

Leukodystrophies commonly begin to manifest in childhood, the so-called "classic expression". However, it is important to recognize that there are late-onset cases, in which disease presentation may be atypical, clinical course often insidious and diagnosis significantly delayed.^3,4^

In this study, we report a case of a previously healthy man initially presenting with neuropsychiatric symptoms and subsequently diagnosed with adrenoleukodystrophy (ALD) at follow-up. The aim of the report is to show the broad spectrum of presentation in adult-onset leukodystrophies, to alert clinicians to the importance of conducting an extensive neurological investigation as part of the assessment of patients presenting with atypical psychiatric disease, and to review clinical and radiological aspects of adult-onset ALD.

## CASE REPORT

A 35-year-old man with no previous known disease presented with a four-year history of changes in mental status and behavior. According to descriptions given by the patient's wife, he developed nervousness, irritability, anxiety, intense episodes of agitation, and aggression. He was referred for psychiatric treatment, having been hospitalized at a mental illness facility and treated with neuroleptics.

One year after the onset of symptoms, the patient suffered an accident at work in a metallurgic factory, resulting in one of his left toes being amputated. At that time he began to present difficulties walking and with balance, initially attributed to the anatomical loss caused by the amputation. The condition worsened and he also began to show cognitive problems such as forgetfulness, disorientation in time and space, and strange behavior at work, which eventually led him to stop working.

After three years of evolution, the clinical picture gradually worsened with severe difficulty walking, leading to a wheelchair-bound state. He also started to have urinary incontinence and speech abnormalities. Cognitive status worsened and he was no longer able to recognize his family and started to have hallucinations. He developed seizures and was put on oxcarbazepine, zolpi-dem and tizanidine.

At first examination of the patient by our service, his neurologic examination revealed dementia with widespread cognitive impairment. The patient was mute and apathetic. Bilateral grasping sign and severe spastic tetraparesis were evident. Tendon reflexes were present, extrinsic and intrinsic ocular motor functions were preserved and fundus examination revealed no papilledema.

Results of laboratory tests were as follows: Serum ACTH with greatly increased value of 1,534 pg/ml (Normal values: 9-52 pg/mL). Erythrocyte sedimentation rate, C-reactive protein, total complement and fractions (C3 and C4), serum ammonia, homocysteine, serum protein electrophoresis, serum cortisol, urinary cortisol levels were all within normal ranges. ANCAs, anti-thyroglobulin, anti-thyroperoxidase, ANA, anti-ENAs, anti-gliadin, anti-endomysium, and rheumatoid factor were all non-reactive. Serological tests for toxoplasmo-sis, cytomegalovirus and Epstein-Barr virus (both IgG and IgM), and HIV were all negative. CSF analysis was normal, except for an increase in gammaglobulin level (14%), associated to an oligoclonal component. Immunoelectrophoresis was normal. Serologies for cysticerco-sis, syphilis, and PCR JC virus were normal.

**Figure 1:**
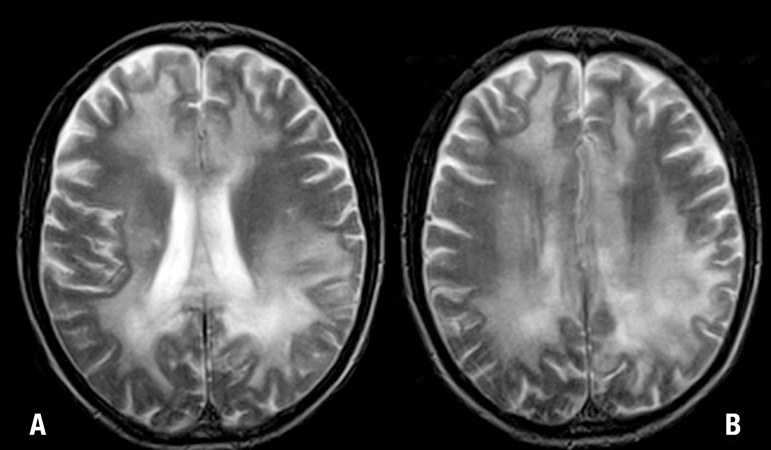
[A, B]. Axial T2-weighted MR images show confluent and symmetric bilateral hyperintense areas in the cerebral white matter, extending to the corpus callosum, especially the splenium. Involvement is more pronounced in parieto-occipital regions.

**Figure 2:**
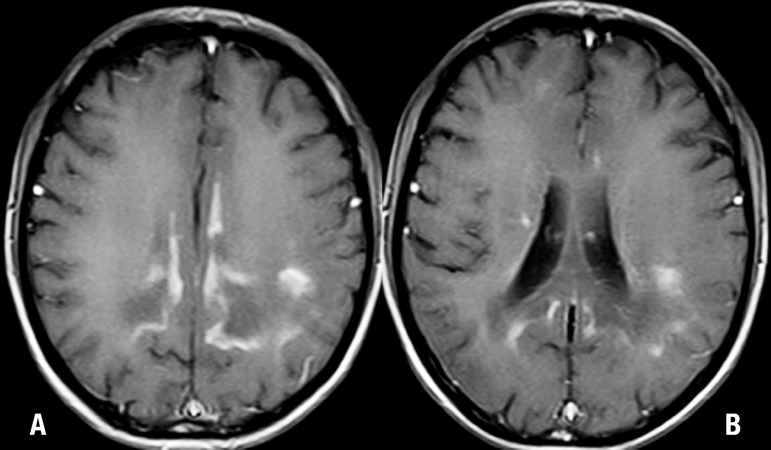
[A, B] Axial post-contrast T1-weighted MR images show intense enhancement in both parietooccipital regions. Enhancement can be appreciated in the so-called "intermediate zone" of the cerebral white matter lesions usually seen in this disease.

A brain MRI disclosed severe, confluent and symmetric changes in the parieto-occipital deep white matter and also in the splenium of the corpus callosum, associated with areas of gadolinium enhancement. Frontal and periventricular deep white matter were affected to a lesser degree (Figures 1 and 2). MRI of the abdomen and pelvis showed no changes, including normal-appearing adrenal glands.

The diagnosis of adult-onset leukodystrophy was suspected and levels of biochemical markers showed increased very long-chain fatty acids in serum (C26:0: 3.66 mMol/L; reference value: <1.3mMol/L). Galacto-cerebrosidase and arylsulfatase levels were normal. No history of similar disease was reported in the family. A diagnosis of adult-onset ALD was determined.

## DISCUSSION

In patients presenting with psychiatric symptoms, cognitive dysfunction, gait abnormalities and white matter lesions on MRI, it is important to rule out leukodystrophies in the differential diagnosis, even in the absence of familial history.^1,2^ The etiological diagnosis depends on an analysis of the pattern and distribution of lesions on imaging studies, presence or absence of associated neurological findings (peripheral neuropathy, optic atrophy, macrocephaly) and any apparent systemic features. Biochemical and/or molecular testing are important ancillary tools.^2^ Common leukoencephalopathies and leukodystrophies that may have adult-onset and initially neuropsychiatric presentation include adreno-leukodystrophy, vitamin B12 deficiency, metachromatic leukodystrophy, multiple sclerosis, Krabbe's disease, and solvent abuse.^2-4^

Adrenoleukodystrophy (ALD) is an X-linked peroxisomal disorder in which very long-chain fatty acids (VLCFA), defined as those having more than 22 carbon chains, accumulate within cells as a result of defective beta-oxidation within the peroxisome. Although ALD is much more frequent in childhood and was previously thought to occur only in this age group, it is now known that ALD can occur over a wide age spectrum and have broad clinical heterogeneity.^3,4^

The childhood-onset ("classic") form is a rapidly progressive neurodegenerative disease, typically emerging between the ages of 3 and 8. Clinical presentation is characterized by progressive attention deficit disorder, followed by intellectual, behavioral, and neurological deterioration. Clinical course is rapidly progressive to a vegetative state and death normally within 2 years.^5,6^ An adolescent-onset form, which is less severe, may present with primary adrenal insufficiency, neurological dysfunction, or psychiatric symptoms. Death usually occurs within 1 to 2 years of cerebral involvement.^5,6^

Adult-onset ALD may have an atypical presentation. Any combination of adrenal, gonadal, neurological, or psychiatric disorders may occur. Most patients will have neurological involvement at some point in the disease course.^7,8^ Although gait disorders and evidence of upper motor neuron involvement are the most frequent initial symptoms, neuropsychiatric presentation has been reported.^9,10^ Neuropsychiatric symptoms include disinhibition, emotional lability, increased spending, hyper-sexuality, loudness, perseveration, irritability and psychosis. As these symptoms are also present in maniac states, the diagnosis of bipolar disorder is sometimes erroneously reached. Although some cases present as typical psychiatric disorders, the refractoriness to treatments, progressive course and presence of neurological abnormalities may alert to an alternative diagnosis.^4^ Neurological findings reported in adult-onset ALD include upper motor neuron signs, urinary incontinence, cerebellar dysfunction, visual field changes, speech disturbances, seizures, nystagmus, and peripheral neuropathy. The presence of cognitive decline is a characteristic that helps to differentiate adult-onset ALD from primary psychiatric disorders. Also gonadal and adrenal dysfunctions are clues to alternative diagnosis.

ALD is caused by mutations in a gene mapped in the X-q28 locus that encodes the protein ALD located in the membrane of peroxisomes. Over 500 mutations have been reported, but there is usually no correlation between genotype and phenotype. This carrier protein belongs to the ABC superfamily and forms part of the "route" through which VLCFA CoA synthetase enzyme moves from the cytosol to the membrane peroxisome. Dysfunction of the enzyme leads to reduced degradation of VLCFAs. Its prevalence is unknown, but there are reports of one case in every 20,000 births. Due to the type of inheritance, ALD is a disease that affects predominantly men, because in women the defective X chromosome is usually permanently inactivated while in an embryonic state. Nevertheless, 20 to 50% of heterozygous females have mild neurological signs, with late-onset in the 4th decade and a prolonged course, which may mimic multiple sclerosis. Only 3% progress with cognitive decline and less than 1% have adrenal in-sufficiency.^7,11^

Pathologically, VLCFAs accumulate in all cells, but particularly in the adrenal cortex, Leydig cells of the testes and in myelin-producing cells within both central and peripheral systems. The levels of VLCFAs in tissues are up to 100 times higher than in normal individuals. Excess VLCFAs leads to their incorporation into cell membrane, which usually only contains fatty acids 1618 carbon chains, causing the membrane to become structurally unstable and have abnormal function. In cells of the adrenal cortex, there is loss of normal response to ACTH, as its membrane receptor expression is reduced. A similar process occurs in Schwann cells and oligodendrocytes. In the CNS, there is initial noninflammatory demyelination, but as the process progresses, inflammatory events resembling multiple sclerosis appear. Release of abnormal lipid antigens likely trigger an inflammatory cascade of events, and there is activation of astrocytes and microglia producing TNF, release of cytokines, and increased expression of Major Histocompatibility Complex Class I T-cell signaling molecules (especially CD8), which leads to the destruction of oligo-dendrocytes.^7,8,12^

Affected white matter of the brain is divided histo-pathologically into three distinct zones: an outermost zone (zone 1), showing active destruction of the myelin sheath and lack of perivascular inflammatory cells; a middle layer zone (zone 2), showing perivascular inflammatory cells and demyelination with preservation of axons; and a central zone (zone 3), showing gliosis and scattered astrocytes with the absence of oligoden-droglia, axons, myelin, and inflammatory cells.^13,14^

As there are no pathognomonic findings associated with X-linked ALD, a high degree of suspicion is important to further investigate the typical features of the disease. Adrenal insufficiency may be associated with electrolyte abnormalities secondary to hypoaldosteronism. Assessment of serum ACTH and baseline cortisol levels (24-hour urine cortisol and A.M. and P.M. serum levels) is important in that, if either is abnormal, an ACTH stimulation test should be performed to evaluate adrenal reserve. Adrenocortical insufficiency in ALD can be life-threatening if not treated, and all patients with ALD should undergo regular reassessment of adrenocorti-cal function if initial results are normal. Patients with ALD often have abnormal auditory evoked potentials and normal visual evoked potentials.^5,15,16^ This pattern is useful diagnostically when differentiating between multiple sclerosis and ALD. The finding of abnormal visual evoked potentials with normal auditory evoked potentials is more suggestive of MS. The definitive diagnosis is reached by detection of elevated VLCFA levels in serum.

MRI has proven crucial in the diagnostic workup of patients with leukoencephalopathies. MRI pattern recognition is a way of systematically analyzing many details on MR images and integrating these into patterns by disease. Schiffmann and Van der Knaap developed a scheme based principally on MRI patterns.^1,2,13^ It involves the differentiation of hypomyelination from other types of white matter pathology; the distinction between confluent and multifocal, isolated white matter abnormalities; the assessment of the predominant localization of confluent white matter abnormalities; and the evaluation of special features such as cysts and their locations, additional gray matter lesions, contrast enhancement, calcium deposits, microbleeds, spinal cord involvement, and evolution over time.^1,13^

MRI can also demonstrate the three zones recognized histologically in ALD.^13,17^ The external zone exhibits active destruction of myelin without inflammation, high signal on T2 and low-to-intermediate signal on T1 (Figures 1A and 1B). The intermediate zone shows signs of active inflammation while MRI shows contrast enhancement on T1 (Figures 2A and 2B). The internal zone is completely demyelinated and exhausted (Figures 1 and 2). It can display cavitation and calcifications best visualized on CT. The white matter involvement often appears symmetrical and bilateral, however, not always after careful evaluation.^13,14,18^ Unlike deep white matter, U fibers and cortex are spared, being most visible on T1-weighted MR.^13,14,18^

Loes et al described five modified MRI patterns in ALD, along with their relative frequencies, ages of affected patients, and patterns of progression on MR imaging. According to this data, the most frequent type is primary involvement of the deep white matter in the parieto-occipital lobes and of the splenium of the corpus callosum (Table 1).^13,17,18^

**Table 1:** Patterns of progression in X-linked Adrenoleukodystrophy on MRI*.

Type	Pattern	Frequency	Age
1	Primary involvement of deep white matter in the parieto-occipital lobes and of the splenium of the corpus callosum. May include lesions of the visual and auditory pathways	66%	Seen mainly in children
2	Primary involvement of the frontal lobe or genu of the corpus callosum	15.5%	Seen mainly in adolescents
3	Primary involvement of the fronto-pontine or corticospinal projection fibers	12%	Seen mainly in adults
4	Primary cerebellar white matter involvement	1%	Seen mainly in adolescents
5	Combined involvement of parieto-occipital and frontal white matter	2.5%	Seen mainly in children

* From Loes DJ, Fatemi A, Melhem ER, et al. Analysis of MRI patterns aids prediction of progression in X- linked adrenoleukodystrophy. Neurology 2003;61:369-374.

MRI can be used not only to diagnose but also to predict disease progression among patients with X-linked ALD.^19^ Because the severity of the inflammatory process has been correlated with rapidity of disease progression, contrast-enhanced T1- weighted spin-echo MR imaging may serve as a marker for the presence and the severity of this inflammatory process.^1,13,19^ On gadolinium-enhanced T1-weighted MR imaging, white matter lesions, especially in the parieto-occipital periventricular area, present enhancement at the peripheral portion corresponding to zone 2, matching the region of active inflammatory demyelination (Figures 2A and 2B).

The enhancement is attributed to a breakdown of the blood-brain barrier resulting from an autoimmune or cytokine-mediated inflammatory process. A lack of enhancement is associated with stable disease in 85-90% of patients.^1^

Although new MRI techniques such as diffusion-weighted imaging and MR spectroscopy have been shown to be clinically useful in patients with childhood cerebral X-linked ALD, conventional brain MRI, with T1- and T2-weighted, and fluid-attenuated inversion recovery (FLAIR) sequences, is widely available and remains a valuable tool for assessing this disease.^1,13,14^

However, MRI pattern recognition does not lead to a specific diagnosis in all patients with white matter abnormalities, because the pattern of MRI abnormalities is not specific in all disorders and the specificity of the pattern is disease and stage-dependent. It is well known that the end stage of most progressive disorders is characterized by the involvement of all cerebral white matter, which may also result in a nonspecific MRI pattern.^1,2^

Despite being a genetically determined disease, some treatments are available for patients with ALD. However, approaches incorporating diets with low levels of VLCFA have failed to reduce plasma concentrations or alter disease progression. As "upregulation" of the VLCFA synthesis also occurs, other dietary treatments have been advocated such as "Lorenzo oil," a combination of oleic acid and erucic acid at a 4:^1^ ratio. These monounsaturated fatty acids inhibit the fatty acid elongation system by competition, reducing the production of VLCFA. Although Lorenzo's oil has been shown to reduce plasma levels of VLCFA, it failed to halt the progression of cerebral forms of ALD, but evidence suggests that presymptomatic patients may benefit from taking it.^20,23^ Studies aimed at blocking the inflammatory process of ALD with immunosuppression using cyclophosphamide, thalidomide and interferon-beta have been unsuccessful. Similarly, treatment with de-hydroepiandrosterone (DHEA) produced no results.^23^ Currently, bone marrow transplantation is being used in patients with no clinical symptoms but evidence of demyelinating lesions on MRI, in an attempt to introduce healthy cells capable of degrading the VLCFA. Bone marrow transplant has proven able to reverse or stabilize the changes on MRI in asymptomatic patients, but was ineffective in patients with neurological symptoms. In view of this evidence, it is crucial that family members be tested and diagnosed early.^24,25^
